# Suture rectopexy *versus* ventral mesh rectopexy for complete full-thickness rectal prolapse and intussusception: systematic review and meta-analysis

**DOI:** 10.1093/bjsopen/zraa037

**Published:** 2021-01-09

**Authors:** H S Lobb, C C Kearsey, S Ahmed, R Rajaganeshan

**Affiliations:** University of Liverpool, Liverpool, UK; St Helen’s and Knowsley Teaching Hospitals NHS Trust; Royal Liverpool and Broadgreen University Hospitals NHS Trust, Liverpool, UK; St Helen’s and Knowsley Teaching Hospitals NHS Trust

## Abstract

**Background:**

This systematic review and meta-analysis aimed to compare recurrence rates of rectal prolapse following ventral mesh rectopexy (VMR) and suture rectopexy (SR).

**Methods:**

MEDLINE, Embase, and the Cochrane Library were searched for studies reporting on the recurrence rates of complete rectal prolapse (CRP) or intussusception (IS) after SR and VMR. Results were pooled and procedures compared; a subgroup analysis was performed comparing patients with CRP and IS who underwent VMR using biological *versus* synthetic meshes. A meta-analysis of studies comparing SR and VMR was undertaken. The Methodological Items for Non-Randomized Studies score, the Newcastle–Ottawa Scale, and the Cochrane Collaboration tool were used to assess the quality of studies.

**Results:**

Twenty-two studies with 976 patients were included in the SR group and 31 studies with 1605 patients in the VMR group; among these studies, five were eligible for meta-analysis. Overall, in patients with CRP, the recurrence rate was 8.6 per cent after SR and 3.7 per cent after VMR (*P* < 0.001). However, in patients with IS treated using VMR, the recurrence rate was 9.7 per cent. Recurrence rates after VMR did not differ with use of biological or synthetic mesh in patients treated for CRP (4.1 *versus* 3.6 per cent; *P* = 0.789) and or IS (11.4 *versus* 11.0 per cent; *P* = 0.902). Results from the meta-analysis showed high heterogeneity, and the difference in recurrence rates between SR and VMR groups was not statistically significant (*P* = 0.76).

**Conclusion:**

Although the systematic review showed a higher recurrence rate after SR than VMR for treatment of CRP, this result was not confirmed by meta-analysis. Therefore, robust RCTs comparing SR and biological VMR are required.

## Teaser

There have been long-term complications associated with synthetic mesh for ventral mesh rectopexy. This double-armed systematic review and meta-analysis aimed to compare recurrence rates of rectal prolapse following ventral mesh rectopexy and suture rectopexy. Limitations include a lack of comparative evidence owing to comparison of historical and current interventions and inconsistent follow-up; however, patient characteristics were consistent. The recurrence rates and safety of suture rectopexy are comparable to those of ventral mesh rectopexy and it could provide a safe alternative. An RCT comparing suture rectopexy and biological ventral mesh rectopexy is required.

Ventral mesh and suture rectopexy in rectal prolapse

## Resumen

**Antecedentes:**

Esta revisión sistemática con metaanálisis tuvo como objetivo comparar la tasa de recidiva del prolapso rectal tras de la rectopexia ventral con malla (*ventral mesh rectopexy*, VMR) o con sutura (*suture rectopexy*, SR).

**Métodos:**

Se realizó una búsqueda de los estudios que notificaran la tasa de recidiva del prolapso rectal completo (*complete rectal prolapse*, CPR) o de la invaginación (*intussusception*, IS) tras SR o VMR en las bases MEDLINE, EMBASE y la Biblioteca Cochrane. Se agruparon los resultados y se compararon por procedimientos; se realizó un análisis de subgrupos comparando los pacientes con CPR e IS en los que se realizó una VMR con malla biológica o sintética. Se efectuó un metaanálisis de los estudios que comparaban SR y VMR. Para evaluar la calidad de los estudios se utilizaron la puntuación del *Methodological Items for Non-Randomised Studies* (MINORS), la escala Newcastle Ottawa y el instrumento de la Colaboración Cochrane.

**Resultados:**

Se incluyeron 22 estudios con 976 pacientes en el grupo SR y 31 estudios con 1.605 pacientes en el grupo VMR. Se incluyeron 5 estudios en el metaanálisis. En general, en los pacientes con CRP, la tasa de recidiva fue del 8,6% tras RS y del 3,7% tras VMR (*P* < 0,05). Sin embargo, en pacientes con IS tratados con VMR, la tasa de recidiva fue del 9,7%. No hubo diferencias en la tasa de recidiva después de VMR utilizando mallas biológicas o sintéticas, tanto en pacientes con CRP (4,1% *versus* 3,6%, *P* = 0,789) como con IS (11,4% *versus* 11,0%, *P* = 0,902). Los resultados del metaanálisis mostraron una heterogeneidad elevada; además, la diferencia en la tasa de recidiva entre los grupos SR y VMR no fue estadísticamente significativa (*P* = 0,76)

**Conclusión:**

Aunque la revisión sistemática puso de manifiesto un mayor número de recidivas en pacientes con CRP tratados con RS que con VMR, este resultado no se confirmó en el metaanálisis. Por lo tanto, se requieren ensayos controlados aleatorizados robustos que comparen la SR y la VMR con mallas biológicas.

## Introduction

Complete rectal prolapse (CRP) is defined as full-thickness protrusion of the rectal wall through the anus^1^. It begins as intussusception (IS) which may or may not be symptomatic^2^. It is a common condition worldwide, which can be difficult to treat successfully and causes significant psychosocial problems for the patient. The aim of treatment is to control the prolapse and relieve incontinence while preventing constipation or obstructive defaecation^3^^,^^4^. Plication of the redundant bowel and/or fixation of the rectum to the sacrum was originally achieved by SR, but has evolved to the use of synthetic, non-absorbable mesh. Recently, mesh rectopexy has been associated with a rise in chronic pain and morbidity^5^ and, as a result, a change to more expensive biological mesh has become the standard^6^.

SR can be performed laparoscopically or via a laparotomy. First described by Cutait^7^ in 1959, SR involves mobilization and fixation of the rectum with a non-absorbable suture. The act of mobilization, suture, and fibrosis keeps the rectum fixed in position as adhesions form, attaching the rectum to the presacral fascia. Although SR is considered a good option for the cure of rectal prolapse/IS in both men and women, some reviews of this procedure noted a better overall clinical outcome in men^8^. This may be due to occult sphincter defects in women, and failure to detect these defects before surgery owing to the lack of routine endoanal ultrasonography in the earlier years of prolapse surgery^9^.

The mesh rectopexy operation was first described by Ripstein^10^ in 1952. Again, after mobilization of the rectum, an anterior sling of synthetic material (either absorbable or non-absorbable) is placed in front of the rectum and sutured to the sacral promontory. The rationale for this is to restore the natural curve of the rectum, which reduces the effect of downward abdominal pressure. The use of a non-elastic synthetic graft provides a firm anterior fascial support even in patients with significant pelvic floor descent, returning the rectum to a normal anatomical position^11^. However, there were long-term complications associated with the use of synthetic mesh for ventral mesh rectopexy (VMR)^5^, so a shift to biological mesh was made.

There is little hard evidence for the use of biological mesh compared with historical techniques. This systematic review and meta-analysis aimed to identify the evidence and compare recurrence rates for SR with those of VMR for patients with CRP or IS.

## Methods

### Data sources and search strategy

Two literature searches were carried out using MEDLINE, Embase, and the Cochrane Library databases. No limitation on study period was set and searches were set for studies on SR and VMR—using either biological or synthetic mesh—using the following criteria: ‘(suture OR sutured) AND rectopex*’ (SR, search 1) and ‘(ventral OR anterior OR mesh) AND rectopex*’ (VMR, search 2). The reference lists from systematic reviews or meta-analyses were reviewed and relevant studies included. Titles and abstracts were screened by two reviewers, and full-text copies were subsequently obtained. Any discrepancies in screening were settled by a third reviewer.

Studies included were randomized and non-randomized studies using open or laparoscopic techniques that reported either symptomatic, anatomical or radiological recurrence of CRP (full-thickness) or IS as outcome measure, as it is the most standardized way of assessing the efficacy of the procedures. Studies were included only if indication and specific data were available for extraction.

Case reports, duplicates, non-English articles, and those reporting follow-up of less than 12 months were excluded. Studies that focused on robotic rectopexy were excluded owing to the novelty of the technique and absence of a SR robotic group. Other exclusion criteria were: SR in children, rectocele, volvulus or mucosal prolapse; and studies that involved posterior rectopexy, concomitant resections, sacrocolpopexy or other abdominal or pelvic procedures directly related to the prolapse or IS. Studies pertaining to VMR were excluded if they used the Ripstein procedure/sling rectopexy, Well’s procedure or the Orr–Loygue procedure, concomitant sacrocolpopexy, or any other concomitant abdominal or pelvic procedures.

Non-randomized studies were assessed for methodological quality using the Methodological Index for Non-Randomized Studies score^12^, and RCTs were assessed independently for risk of bias using the Cochrane Collaboration tool^13^, by two reviewers; discrepancies were discussed and resolved mutually.

### Data extraction and outcome measures

The following information was extracted: study design, title, authors, publication year, study type, number of patients undergoing rectopexy, population characteristics, type of mesh used (VMR), duration of follow-up, and number of patients with recurrence of CRP or IS (primary outcome). Secondary outcomes included incontinence and constipation data, and postoperative complications reported by the studies. Secondary procedures and secondary recurrence were excluded, and partial recurrence was not considered an outcome of interest. In calculation of the complication rate, only studies that reported complications were included in the denominator.

Constipation and incontinence data varied among studies, as various scoring methods (Cleveland and Wexner scores, and Faecal Incontinence Severity Index) were reported. Data extraction for these outcomes included type of scoring system used if available, values from each scoring system, raw figures for patients with incontinence or constipation before and after operation if available, and whether the study reported a change in symptoms to be statistically significant.

### Statistical analysis

Data extracted from the studies were pooled for the overall rates of recurrence and complications. The significance of recurrence and complication rates was assessed using Pearson’s χ^2^ test in SPSS^®^ (IBM, Armonk, New York, USA); *P*  < 0.050 was considered statistically significant. Constipation and incontinence data were considered for qualitative analysis. Randomized and non-randomized studies comparing SR and VMR were eligible for meta-analysis and statistical comparison of recurrence rates. The quality of non-randomized studies was assessed using the Newcastle–Ottawa Scale^14^ and risk of bias of randomized studies using the Cochrane Collaboration tool^13^. Meta-analysis was performed using Review Manager 5.3 (Nordic Cochrane Centre, Copenhagen Denmark). Risk ratio was the effect measure used (with 95 per cent confidence interval) and statistical heterogeneity was assessed using the *I*^2^ test. A random-effects model was to be used if heterogeneity was high (*I*^2^ over 50 per cent) and a fixed-effect model if heterogeneity was low. Results were represented visually in a forest plot. *P* < 0.050 indicated statistical significance.

## Results

**Fig. 1:**
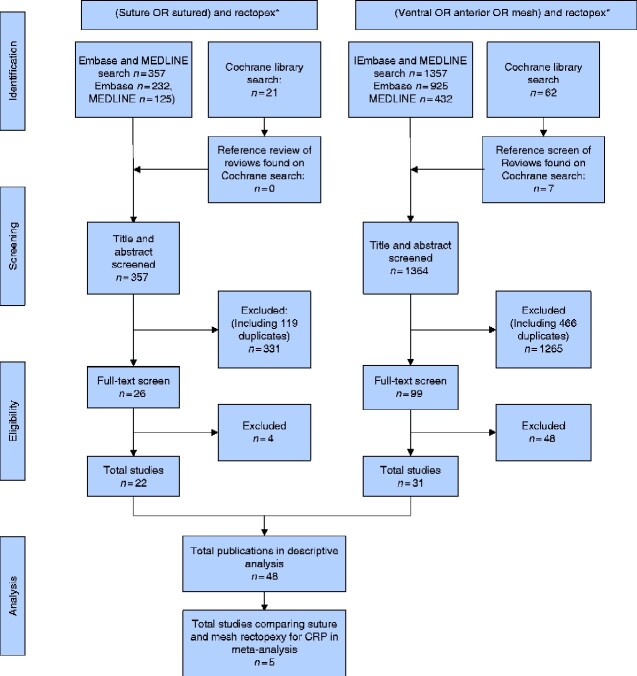
PRISMA flow diagram showing selection of studies for review CRP, complete rectal prolapse.

Of 378 citations retrieved from the SR search, 22^8^^,^^9^^,^^15–34^ were included in the review including 976 patients. Of 1419 citations retrieved from the VMR search, 31 studies^15^^,^^21^^,^^23^^,^^27^^,^^30^^,^^35–60^ were included in analysis reporting on 1608 patients with CRP and 399 patients with IS (*Fig. 1*). All studies in the SR group included patients with CRP. Data for CRP and IS were therefore compared separately. Studies and their characteristics are summarized in *Tables 1* and *2*.

**Table 1:** Characteristics of studies of suture rectopexy

Reference	Study type	No. of patients	**Age (years)** ^*^	% women	Follow-up method	**Duration of follow-up (months)** ^*^	MINORS score	Cochrane Collaboration tool score
Benoist *et al*.^15^	Retrospective	16	76.2^†^	100	Clinical examination	24^†^	15 of 24	–
Blatchford *et al*.^16^	Retrospective	42	61^‡^	88	Office visits/telephone interviews	28	11 of 16	–
Briel *et al*.^9^	Retrospective	24	71	88	Hospital records and prospective telephone interview	67	10 of 16	–
Bruch *et al*.^17^	Prospective	32	62^†^	94	Clinic appointment, continence score, anorectal manometry	30^†^	12 of 16	–
Chaudhry Vsm^18^	Prospective	36	43.5^†^	72	Not-specified	12	11 of 16	–
De Oliviera *et al*.^19^	Retrospective	16	82	88	Examination/patient complaint	29^†^	16 of 24	–
Foppa *et al*.^20^	Prospective	172	62	97.2	Telephone interview and office appointment	162	13 of 16	–
Gleditsch *et al*.^21^	Retrospective	49	72	83	Interview, endoscopy and examination	84	15 of 24	–
Heah *et al*.^22^	Retrospective	25	72^‡^	88	Outpatient appointment or telephone review	26	11 of 16	–
Hidaka *et al*.^23^	RCT	30	48.5	90	Clinical examination	72	–	3 unclear 4 low risk
Kellokumpu *et al*.^24^	Prospective	16	57	91	Appointment at hospital and endoscopy	24	14 of 24	–
Kessler *et al*.^8^	Retrospective	28	51.5	84	Telephone interview	33	9 of 16	–
Khanna *et al*.^25^	Prospective	65	n.a.	n.a.	n.a.	65	10 of 16	–
Liyanage *et al*.^26^	Prospective	70	37	30	Outpatient appointment or telephone/postal review	56	12 of 16	–
Luglio *et al*.^27^	RCT	11	68	100	Questionnaire, endoscopy and defaecography	12	–	5 unclear 2 low risk
McKee *et al*.^28^	RCT	8	70^†^	50	Examination	20	–	5 unclear 2 low risk
Novell *et al*.^29^	RCT	32	76	98	Outpatient appointment or telephone/postal review	50	–	3 unclear 3 low risk 1 high risk
Raftopoulos *et al*.^30^	Retrospective	163	53	70.9	Patient data	43	16 of 24	–
Sahoo *et al*.^31^	Retrospective	32	42.5^†^	n.a.	Hospital records	12	15 of 24	–
Senapati *et al*.^32^	RCT	35	58^†^	84	Clinic appointment and questionnaire	36	–	4 unclear 3 low risk
Wilson *et al*.^33^	Prospective	59	72	99	Telephone interview	48	9 of 16	–
Yasukawa *et al*.^34^	Case series	15	72.5^†^	93	Telephone interview	16	10 of 16	–

*Values are median unless indicated otherwise; values are ^†^mean and ^‡^average. MINORS, Methodological Index for Non-Randomized Studies; n.a., not available.

**Table 2:** Characteristics of studies of mesh rectopexy

Reference		Study type	No. of patients		**Age (years)** ^*^	% women	Follow-up method	**Duration of follow-up (months)** ^*^	Type of mesh	MINORS score	Cochrane Collaboration tool score
			CRP	IS							
Albayati *et al*.^35^	Retrospective		9	42	57	100	Questionnaire and telephone call	22	Biological	8 of 16	–
Benoist *et al*.^15^	Retrospective		14	0	76.2^†^	100	Clinical examination	24^†^	n.a.	15 of 24	–
Bjerke and Mynster^36^	n.a.		40	0	83	100	n.a.	18	Synthetic	7 of 16	–
Boons *et al*.^37^	Prospective		65	0	72	92	Clinic appointment and telephone call	19	Synthetic	11 of 16	–
Brunner *et al*.^38^	Prospective		13	0	64.7^†^	94	Clinical examination and questionnaire	29	Biological	11 of 16	–
Byrne *et al*.^39^	Prospective		126	0	56.2^†^	n.a.	Telephone interview and contacted GP	60	Synthetic	10 of 16	–
Chandra *et al*.^40^	Prospective		15	0	50	60	Examination and long-term telephone consultation	22	Synthetic	10 of 16	–
Collinson *et al*.^41^	Prospective		0	75	58	92	Outpatient clinic	12	Synthetic	11 of 16	–
Consten *et al*.^42^	Retrospective		242	0	55.8^†^	94.6	Outpatient clinic	40	Synthetic	11 of 16	–
D’Hoore and Penninckx^43^	Prospective		109	0	F: 50 M: 32	91.7	n.a.	n.a.	Synthetic	9 of 16	–
Emile *et al*.^44^	RCT		25	0	39.7^†^	62	Consultation and examination	18^†^	Synthetic	–	3 unclear 4 low risk
Faucheron *et al*.^45^	Prospective		175	0	58^†^	90.3	Examination	74	Synthetic	12 of 16	–
Franceschilli *et al*.^46^	Prospective		0	98	63^†^	100	Outpatient clinic	20	Biological	13 of 16	–
Gleditsch *et al*.^21^	Retrospective		22	0	72	83	Interview, endoscopy, and examination	29	Biological or synthetic	16 of 24	–
Gosselink *et al*.^47^	Prospective		41	50	CRP: 63 IS: 59	93	Questionnaire and outpatient clinic	12	Synthetic	10 of 16	–
Hidaka *et al*.^23^	RCT		34	0	56.5	91	Clinical examination	72	n.a.	–	3 unclear 4 low risk
Hiltunen and Matikainen^48^	Prospective		54	0	53^†^	82	Outpatient clinic	36	Synthetic	12 of 16	–
Lechaux *et al*.^49^	Retrospective		35	0	53	92	Clinical review and postal questionnaire	36	Synthetic	9 of 16	–
Luglio *et al*.^27^	RCT		20	0	68	100	Questionnaire, endoscopy and defaecography	12	n.a.	–	5 unclear 2 low risk
Madbouly and Youssef^50^	Retrospective		41	0	55^†^	81	Clinical review and postal questionnaire	46^†^	n.a.	18 of 24	–
Maggiori *et al*.^51^	Prospective		20	0	64^†^	88	Examination or telephone consultation	42	Synthetic	10 of 16	–
Mantoo *et al*.^52^	Prospective		23	0	62^†^	n.a.	Outpatient clinic	16	Synthetic	19 of 24	–
Mehmood *et al*.^53^	Prospective		34	0	59	94	Questionnaire	12	Biological	17 of 24	–
Ogilvie *et al*.^54^	Prospective		33	0	72.3^†^	100	Clinic/examination	16	Synthetic	16 of 24	–
Owais *et al*.^55^	Prospective		18	50	34.5	0	Questionnaire	42	Mostly synthetic	9 of 16	–
Portier *et al*.^56^	Prospective		0	40	60.6^†^	100	Outpatient clinic, examination and questionnaire	22^†^	Synthetic	9 of 16	–
Raftopoulos *et al.*^30^	Retrospective		125	0	53	70.9	Examination in outpatient clinic or telephone interview	43	Synthetic	16 of 24	–
Randall *et al*.^57^	Prospective		190	0	69	87.4	Appointment	29	Synthetic	11 of 16	–
Tsunoda *et al*.^58^	Prospective		0	44	76	100	Questionnaires and proctography	26	Synthetic	9 of 16	–
Tsunoda *et al*.^59^	Retrospective		58	0	80	90	Outpatient clinic, telephone interview, mail questionnaire	49	Synthetic	10 of 16	–
Wahed *et al*.^60^	Prospective		27	0	62	95	examination and proctogram	12	Biological	11 of 16	–

*Values are median unless indicated otherwise; values are

^†^mean. CRP, complete rectal prolapse; IS, intussusception; MINORS, Methodological Index for Non-Randomized Studies; n.a., not available.

In the VMR group, 27 of the 31 studies reported on patients with a median or mean age of more than 50 years, and in 25 studies the study population included more than 80 per cent women. Similarly, in the SR group, median or mean age exceeded 50 years in 17 of 21 studies in which age was reported, and in 16 reports women comprised more than 80 per cent of the included patients.

### Follow-up and recurrences

Follow-up ranged from 12 to 74 months in the VMR group and from 12 to 162 months in the SR group; it was reported using median values in 41 studies and as a mean value in seven. Follow-up data were missing from one VMR study^43^, although this was an update of a previous publication that reported a median follow-up of 61 months^61^. Among patients treated for CRP, the recurrence rate was 8.8 per cent in in the SR group and 3.8 per cent in the VMR group (*P <* 0.001) (*Table 3*). However, among 402 patients with IS treated using VMR, the recurrence rate was 9.7 per cent.

**Table 3:** Recurrences according to surgical approach

Reference	No. of patients	No. of recurrences (%)
**Suture rectopexy**	976	86 (8.8)
Benoist *et al*.^15^	16	0 (0)
Blatchford *et al*.^16^	42	1 (2)
Briel *et al*.^9^	24	0 (0)
Bruch *et al*.^17^	32	0 (0)
Chaudhry Vsm^18^	36	1 (3)
De Oliviera *et al*.^19^	16	2 (13)
Foppa *et al*.^20^	172	30 (17.4)
Gleditsch *et al*.^21^	49	15 (31)
Heah *et al*.^22^	25	0 (0)
Hidaka *et al*.^23^	30	7 (23)
Kellokumpu *et al*.^24^	16	2 (13)
Kessler *et al*.^8^	28	2 (7)
Khanna *et al*.^25^	65	0 (0)
Liyanage *et al*.^26^	70	5 (7)
Luglio *et al*.^27^	11	3 (27)
McKee *et al*.^28^	8	0 (0)
Novell *et al*.^29^	32	1 (3)
Raftopoulos *et al*.^30^	163	1 (0.1)
Sahoo *et al*.^31^	32	0 (0)
Senapati *et al*.^32^	35	9 (26)
Wilson *et al*.^33^	59	6 (10)
Yasukawa *et al*.^34^	15	1 (7)
**Ventral mesh rectopexy**		
Recurrence of complete rectal prolapse	1608	61 (3.8)
Albayati *et al*.^35^	9	1 (11)
Benoist *et al*.^15^	14	0 (0)
Bjerke and Mynster^36^	40	2 (5)
Boons *et al*.^37^	65	1 (2)
Brunner *et al*.^38^	13	1 (8)
Byrne *et al*.^39^	126	5 (4.0)
Chandra *et al*.^40^	15	0 (0)
Consten *et al*.^42^	242	13 (5.4)
D’Hoore and Penninckx^43^	109	4 (3.7)
Emile *et al*.^44^	25	2 (8)
Faucheron *et al*.^45^	175	2 (1.1)
Gleditsch *et al*.^21^	22	3 (14)
Gosselink *et al*.^47^	41	1 (2)
Hidaka *et al*.^23^	34	3 (9)
Hiltunen and Matikainen^48^	54	1 (2)
Lechaux *et al*.^49^	35	1 (3)
Luglio *et al*.^27^	20	1 (5)
Madbouly and Youssef^50^	41	1 (2)
Maggiori *et al*.^51^	20	0 (0)
Mantoo *et al*.^52^	23	2 (9)
Mehmood *et al*.^53^	34	0 (0)
Ogilvie *et al*.^54^	33	5 (15)
Owais *et al*.^55^	18	0 (0)
Raftopoulos *et al.*^30^	125	9 (7.2)
Randall *et al*.^57^	190	1 (0.5)
Tsunoda *et al*.^59^	58	1 (2)
Wahed *et al*.^60^	27	1 (4)
Recurrence of intussusception	399	39 (9.8)
Albayati *et al*.^35^	42	2 (5)
Collinson *et al*.^41^	75	4 (5)
Franceschilli *et al*.^46^	98	14 (14.3)
Gosselink *et al*.^47^	50	3 (6)
Owais *et al*.^55^	50	0 (0)
Portier *et al*.^56^	40	1 (3)
Tsunoda *et al*.^58^	44	15 (34)

Values in parentheses are percentages. *P* < 0.001, suture rectopexy *versus* ventral mesh rectopexy for complete rectal prolapse (Pearson’s χ^2^ test).

Twenty-one studies of VMR reported the use of synthetic mesh, whereas the use of biological mesh was reported in seven (*Table 4*). The remaining VMR studies either did not report the type of mesh used, or used both types and did not specify which mesh was used in patients who had recurrence. Synthetic mesh was used in 1362 patients with CRP across 17 studies, of whom 49 (3.6 per cent) had a recurrence, and in 209 patients with IS across four studies, of whom 23 (11.0 per cent) developed recurrence. Biological mesh was used in 97 patients with CRP across five studies, of whom four (4.1 per cent) had a recurrence, and in 140 patients with IS across two studies, of whom 16 (11.4 per cent) developed recurrence. There was no significant difference in recurrence rates between synthetic or biological mesh for CRP (*P* = 0.789) or IS (*P* = 0.902),

**Table 4:** Comparison between biological and synthetic mesh for mesh rectopexy

Type of mesh	No. of studies		Recurrence		
	CRP	IS	CRP	IS	Total
Biological	5	2	4 of 97 (4)	16 of 140 (11.4)	20 of 237 (8.4)
Synthetic	17	4	49 of 1362 (3.6)	23 of 209 (11.0)	72 of 1571 (4.6)
*P* ^*^			0.789	0.902	

Values in parentheses are percentages. CRP, complete rectal prolapse; IS, intussusception.

*Pearson’s χ^2^ test.

### Constipation and incontinence

In the VMR group, 27 studies reported data on incontinence and 21 found a statistically significant improvement after surgery (*Table 5*). In the SR group, 17 studies reported data on incontinence, eight of which found a statistically significant improvement after operation. One study^54^ in the VMR group and five^19^^,^^25^^,^^28^^,^^29^^,^^31^ in the SR group did not report statistical significance testing, but suggested an improvement in incontinence. No studies reported an overall worsening of incontinence.

**Table 5:** Constipation and incontinence reported in included studies

	Incontinence		Constipation	
	Method of measurement	Statistically significant improvement	Method of measurement	Statistically significant improvement
**Suture rectopexy (CRP)**				
Benoist *et al*.^15^	Raw figures	n.s.	Raw figures	n.s.
Blatchford *et al*.^16^	Graded 0–4 and raw figures	Yes	Raw figures	No, significantly worse constipation
Briel *et al*.^9^	Browning and Parks	Unclear	n.a.	n.a.
Bruch *et al*.^17^	Luebeck continence score	Yes	Raw figures	Yes, but includes some patients who had resection rectopexy
Chaudhry Vsm^18^	Browning and Parks	Yes	Raw figures	n.s. but 9 of 15 patients improved
De Oliviera *et al*.^19^	Wexner score	n.s. but 9 of 11 patients improved	n.a.	n.a.
Foppa *et al*.^20^	Wexner score	Yes	Wexner score	No
Gleditsch *et al*.^21^	n.a.	n.a.	n.a.	n.a.
Heah *et al*.^22^	Browning and Parks	Yes	Raw figures	No
Hidaka *et al*.^23^	CCIS	n.s.	CCCS, PAC-QOL, PAC-SYM	n.s.
Kellokumpu *et al*.^24^	Browning and Parks	Yes	Numerical symptom score	Yes
Kessler *et al*.^8^	n.a.	n.a.	n.a.	n.a.
Khanna *et al*.^25^	Raw figures	n.s. but 12 of 16 patients improved	Raw figures	n.s. but 5 of 6 patients improved
Liyanage *et al*.^26^	Wexner score, and Browning and Parks	Yes, but includes some patients who had resection rectopexy	Rome II criteria	n.s.
Luglio *et al*.^27^	Wexner score	n.s.	Wexner score	n.s.
McKee *et al*.^28^	Saline solution infusion test (raw figures)	n.s. but only 1 of 5 patients had postoperative incontinence	Raw figures	No
Novell *et al*.^29^	Browning and Parks, raw figures	n.s. but 7 of 10 regained continence to solid and liquid	n.a.	n.a.
Raftopoulos *et al*.^30^	n.a.	n.a.	n.a.	n.a.
Sahoo *et al*.^31^	Wexner score	n.s. but 19 of 21 patients improved	Wexner score	n.s. but 11 of 18 patients improved
Senapati *et al*.^32^	Vaizey score	Yes	n.a.	n.a.
Wilson *et al*.^33^	n.a.	n.a.	n.a.	n.a.
Yasukawa *et al*.^34^	n.a.	n.a.	Raw figures	n.s. but 4 of 10 patients improved
**Ventral mesh rectopexy (CRP and IS)**				
Albayati *et al*.^35^ (CRP)	Raw figures	No	Raw figures	No
Albayati *et al*.^35^ (IS)	Raw figures	Yes	Raw figures	Yes
Benoist *et al*.^15^ (CRP)	Raw figures	n.s.	Raw figures	n.s.
Bjerke and Mynster^36^ (CRP)	Wexner score	Yes	Laxatives use (raw figures)	No
Boons *et al*.^37^ (CRP)	FISI	Yes	Wexner score	Yes
Brunner *et al*.^38^ (CRP)	CCIS	Yes	CCIS	Yes
Byrne *et al*.^39^ (CRP)	St Mark’s incontinence score	Yes	Visual analogue constipation score and perceived change (raw figures)	No
Chandra *et al*.^40^ (CRP)	FISI	Yes	Wexner score	Yes
Collinson *et al*.^41^ (IS)	FISI	Yes	Wexner score	Yes
Consten *et al*.^42^ (CRP)	Browning and Parks	Yes, but includes patients with IS/symptomatic rectocele not included in recurrence data	Rome II criteria	n.s. but 50 of 82 improved
D’Hoore and Penninckx^43^ (CRP)	n.a.	n.a.	n.a.	n.a.
Emile *et al*.^44^ (CRP)	Wexner score	Yes	Wexner score	n.s. but large improvement in Wexner score
Faucheron *et al*.^45^ (CRP)	n.a.	n.a.	n.a.	n.a.
Franceschilli *et al*.^46^ (IS)	FISI	Yes	Wexner score	Yes
Gleditsch *et al*.^21^ (CRP)	n.a.	n.a.	n.a.	n.a.
Gosselink *et al*.^47^ (CRP)	FISI	Yes	Wexner score	Yes
Gosselink *et al*.^47^ (IS)	FISI	Yes	Wexner score	Yes
Hidaka *et al*.^23^	CCIS	n.s.	CCCS, PAC-QOL, PAC-SYM	n.s.
Hiltunen and Matikainen^48^ (CRP)	Raw figures	Yes	n.a.	n.a.
Lechaux *et al*.^49^ (CRP)	Wexner score	No	Wexner score	n.s.
Luglio *et al*.^27^ (CRP)	Wexner score	n.s.	Wexner score	n.s.
Madbouly and Youssef^50^ (CRP)	Wexner score	Yes	Wexner score	Yes
Maggiori *et al*.^51^ (CRP)	Wexner score	Yes	Rome II criteria	n.s. but 13 of 18 improved
Mantoo *et al*.^52^ (CRP)	Wexner score	Unclear	ODS score	n.s. but improvement in mean score
Mehmood *et al*.^53^ (CRP)	FISI	Yes	Wexner score	Yes
Ogilvie *et al*.^54^ (CRP)	CCIS	n.s. but large improvement in mean CCIS scores	n.a.	n.a.
Owais *et al*.^55^ (IS and CRP)	CCIS	Yes	ODS score	Yes
Portier *et al*.^56^ (IS)	CCIS	Yes	Raw figures	n.s. but 13 of 20 improved
Raftopoulos *et al.*^30^	n.a.	n.a.	n.a.	n.a.
Randall *et al*.^57^ (CRP)	CCIS	Yes	n.a.	n.a.
Tsunoda *et al*.^58^ (IS)	FISI	Yes	CSS	Yes
Tsunoda *et al*.^59^ (CRP)	FISI	Yes	CSS	Yes
Wahed *et al*.^60^ (CRP)	Wexner score	Yes	Wexner score	Yes

CRP, complete rectal prolapse; n.s., not stated; n.a., not available; CCIS, Cleveland Clinic Incontinence Score; CCIS, Cleveland Clinic Constipation Score; PAC-QOL, Patient Assessment of Constipation Quality of Life questionnaire; PAC-SYM, Patient Assessment of Constipation Symptom score; IS, intussusception; FISI, Faecal Incontinence Severity Index; ODS, obstructive defaecation syndrome; CSS, Constipation Scoring System.

In the VMR group, 24 studies reported data on constipation and 14 found a statistically significant improvement after operation (*Table 5*). In the SR group, 14 studies reported data on constipation, two of which found a statistically significant postoperative improvement. Nine further studies^18^^,^^25^^,^^31^^,^^34^^,^^42^^,^^44^^,^^51^^,^^52^^,^^56^ did not report statistical significance testing, but suggested an improvement in constipation. One study showed a significant worsening of constipation after SR.

Of five studies that compared SR and VMR, three^15^^,^^23^^,^^27^ reported a comparison of incontinence and constipation (*Table 6*). Regarding incontinence, two studies found no statistical difference between VMR and SR, although one^27^ reported a significant difference favouring VMR. With respect to constipation, two studies^23^^,^^27^ reported a statistical difference between VMR and SR, both favouring VMR; however, one of these studies^27^ included some patients who had concurrent sigmoid resection with SR. The third study^15^ did not perform significance testing on constipation data, but reported a similar worsening after VMR and SR.

**Table 6:** Constipation and incontinence in comparative studies

Reference	Incontinence		Constipation	
	Method of measurement	Results	Method of measurement	Results
Benoist *et al.*^15^	Raw figures	No significant difference	Raw figures	n.s., but similar worsening in constipation following VMR and SR
Hidaka *et al.*^23^	CCIS	No significant difference	ODS score, CCCS, PAC-QOL, PAC-SYM	VMR statistically better than SR in all parameters
Luglio *et al.*^27^	Wexner score	VMR statistically better than SR	Wexner score, Rome III criteria	VMR statistically better than SR; however, some patients who had resection rectopexy were included in SR group

n.s., Not stated; VMR, ventral mesh rectopexy; SR, suture rectopexy; CCIS, Cleveland Clinic incontinence Score; CCCS, Cleveland Clinic Constipation Score; PAC-QOL, Patient Assessment of Constipation Quality of Life questionnaire; PAC-SYM, Patient Assessment of Constipation Symptom score.

### Complications

Twelve studies in the SR group reported complications, including 616 patients with 54 complications overall (8.8 per cent) (*Table 7*). Twenty-two VMR studies reported complications including 1232 patients and 97 complications overall (7.9 per cent) (*P* = 0.509 for SR *versus* VMR). The most common postoperative complications reported were surgical-site infection after SR (1.9 per cent) and urinary tract infection after VMR (2.4 per cent).

**Table 7:** Summary of complications by procedure

	Suture rectopexy (*n* = 616)	Mesh rectopexy (*n* = 1232)
Atelectasis	0 (0)	1 (0.1)
Atrial fibrillation	1 (0.2)	0 (0)
Bladder injury	0 (0)	1 (0.1)
Bleeding from port site	1 (0.2)	0 (0)
Deep vein thrombosis	4 (0.6)	0 (0)
Enterocutaneous fistula	0 (0)	0 (0)
Faecal impaction	0 (0)	1 (0.1)
Fluid overload	0 (0)	1 (0.1)
Haematoma	1 (0.2)	10 (0.8)
Hypertension	1 (0.2)	0 (0)
Incisional/port-site hernia	3 (0.5)	7 (0.6)
Infective diarrhoea	2 (0.3)	0 (0)
Intestinal obstruction	4 (0.6)	2 (0.2)
Lumbar discitis	0 (0)	1 (0.1)
Myocardial infarction	0 (0)	1 (0.1)
Non-specific bleeding	1 (0.2)	1 (0.1)
Non-specific infection	0 (0)	2 (0.2)
Pain	0 (0)	6 (0.5)
Pelvic abscess	2 (0.3)	0 (0)
Pelvic collection	1 (0.2)	0 (0)
Perforated bowel	2 (0.3)	3 (0.2)
Peritonitis	1 (0.2)	0 (0)
Pneumonia	3 (0.5)	3 (0.2)
Presacral vein injury	2 (0.3)	0 (0)
Prolonged ileus	1 (0.2)	12 (1.0)
Pulmonary oedema	0 (0)	0 (0)
Respiratory failure	0 (0)	0 (0)
Retrograde ejaculation	0 (0)	0 (0)
Sphincterismus	0 (0)	0 (0)
Subcutaneous emphysema	1 (0.2)	3 (0.2)
Surgical-site infection	12 (1.9)	5 (0.4)
Upper gastrointestinal bleed	0 (0)	0 (0)
Ureteric injury	2 (0.3)	1 (0.1)
Urinary incontinence	0 (0)	2 (0.2)
Urinary retention	6 (1.0)	4 (0.3)
Urinary tract infection	3 (0.5)	29 (2.4)
Wound abscess	0 (0)	1 (0.1)
Total	54 (8.8)	97 (7.9)^*^

Values in parentheses are percentages.

*P  = 0.509 *versus* suture rectopexy (Pearson’s χ^2^ test).

### Meta-analysis

Of the 48 studies, five (2 RCTs and 3 non-randomized studies) compared recurrence of CRP after SR *versus* VMR and were therefore eligible for meta-analysis (*Table 8*). Of the randomized studies, risk of bias assessed using the Cochrane Collaboration tool was considered to be low in one^23^ and unclear in the other^27^ . Of the three non-randomized studies, one was considered to be of fair quality (4 of 7)^15^ and the other two^21^^,^^30^ of high quality (7 of 7 and 6 of 7) (*Tables S1* and *S2*).

**Table 8:** Characteristics of studies included in meta-analysis

Reference	Study design	No. of patients		Comparators	Inclusion criteria	Exclusion criteria	Method of measuring recurrence	Outcome measures	**Duration of follow-up (months)** ^*^
		SR	VMR						
Benoist *et al*.^15^	Retrospective, observational (1993–1995)	16	14	VMR *versus* SR with and without sigmoid resection	Patients who had surgery for full-thickness rectal prolapse	Patients who had a hand-assisted procedure	Clinical examination or long-term telephone interview	Complications, constipation, incontinence, recurrence	24^†^
Gleditsch *et al*.^21^	Retrospective, observational (1998-2017)	49	22	Laparoscopic posterior SR *versus* VMR	Patients who had surgery for external rectal prolapse	Patients with internal rectal prolapse	Clinical examination and endoscopy	Complications, recurrence	SR: 84 VMR: 29
Hidaka *et al*.^23^	RCT (2006–2014)	30	34	Laparoscopic posterior SR *versus* VMR	Patients with rectal prolapse	n.a.	Clinical examination and questionnaires	CCCS, CCIS, ODS score, PAC-QOL, PAC-SYM, prolapse recurrence, mesh complications	72
Luglio *et al*.^27^	RCT (2013–2015)	11	20	VMR *versus* SR	ODS, persistent bleeding, full-thickness rectal prolapse, squeeze pressure > 60 mmHg	n.a.	Questionnaire, endoscopy, and defaecography	Rome III criteria, Wexner incontinence and constipation scores, endoscopy and defaecography	12
Raftopoulos *et al*.^30^	Retrospective, observational (1979–2001)	122	117	Mobilization only, mobilization–resection–pexy, or mobilization–pexy Means of access: open or laparoscopic Rectopexy method: suture or mesh	Patients who had abdominal surgery for full-thickness rectal prolapse	Patients without follow-up	Physical examination in outpatient clinic or telephone interview	Recurrence	43

*Values are median unless indicated otherwise;

^†^values are mean. SR, suture rectopexy; VMR, ventral mesh rectopexy; n.a., not available; CCIS, Cleveland Clinic Incontinence Score; CCCS, Cleveland Clinic Constipation Score; ODS, obstructive defaecation syndrome; PAC-QOL, Patient Assessment of Constipation Quality of Life questionnaire; PAC-SYM, Patient Assessment of Constipation Symptom score.

Length of follow-up varied between the studies ranging from 12 to 84 months. The method of assessing recurrence of CRP was robust in all five studies, which reported the use of clinical examination with or without questionnaires, endoscopy or defaecography.

**Fig. 2:**
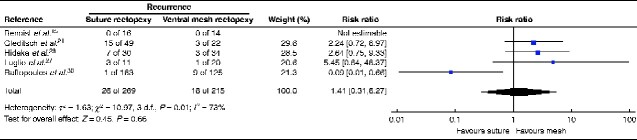
Forest plot of recurrence after suture rectopexy *versus* ventral mesh rectopexy for complete rectal prolapse A Mantel–Haenszel random-effects model was used for meta-analysis. Risk ratios are shown with 95 per cent confidence intervals.

Across the five studies, 269 patients had SR, of whom 26 had a recurrence (9.7 per cent) and 215 had VMR, of whom 16 developed recurrence (7.4 per cent). Statistical heterogeneity was high (*I*^2^ = 73 per cent) and the difference in recurrence rates was not statistically significant (*P* = 0.66; 3 d.f.) (*Fig. 2*).

## Discussion

The concept of fixing the rectum to the sacrum has been a mainstay in the treatment of rectal prolapse for 35 years. The original Orr–Loygue procedure, which involves fully mobilizing the rectum circumferentially down to the levator ani muscle, and fixing an anterior and posterior mesh from the sacrum to the anterolateral rectal wall, has been modified over the years^62^. The D’Hoore modified method performed laparoscopically demands only that Denonvilliers fascia is dissected around the anterior rectal wall and a single mesh is sutured to the anterior aspect of the distal rectum. Owing to possible complications of neurological damage, posterior dissection is avoided in the modified procedure and is limited only to clear the sacral promontory sufficiently for mesh fixation to the periosteum^43^.

When considering synthetic mesh as a material for rectal fixation, the tensile strength of most synthetic materials usually exceeds the physiological demand. This excess tensile strength can lead to an increased local inflammatory response and loss of elasticity of the mesh. On the other hand, biological meshes are made from human, bovine or porcine tissue that has been decellularized to leave a collagen matrix for native tissue to infiltrate. The characteristics of each material are unique and depend on the tissue source, the method used to remove the cells, and the method of sterilization. However, it is in terms of the safety profile that biological mesh has become superior to synthetic mesh^63^.

Anecdotally, the complication rate associated with biological mesh appears to be lower than that for synthetic mesh, probably related to its lower tensile strength, but its cost for VMR remains a problem. Before the development of VMR, simple sutures were used for rectopexy. Historically, there have been numerous subtle variations of this technique, but the general consensus was to use two or three non-absorbable sutures for fixation of the rectum to the sacrum^7^.

This review aimed to compare recurrence rates following CRP and IS. However, the SR group did not include any patients with IS and so a subgroup analysis was performed in the VMR group. The recurrence rate was higher after SR than VMR in patients treated for CRP, whereas the subgroup analysis of patients who underwent VMR showed higher rates in patients with IS than those with CRP.

Given that biological VMR is the current standard treatment for CRP and IS, it is important to note that, of the seven studies (237 patients) that reported the use of biological mesh, the recurrence rate was similar to that of SR (recurrence rate of IS and CRP combined 8.4 per cent after VMR *versus* 8.8 per cent for CRP after SR) (*Tables 3* and *4*) The small number of studies reporting recurrence following biological VMR highlights the need for further research. Comparison of the two groups using meta-analysis showed no statistical difference in recurrence of CRP between synthetic VMR and SR.

It appears that constipation and incontinence improved more after VMR. However, poor consistency of reporting, variation in methods of measuring constipation and incontinence across studies, and varying interpretation of these methods made comparison of studies challenging in this study and reduces the reliability of these results.

Few studies reported postoperative complications and, although complication rates were similar after both procedures, heterogeneity between studies will have had a considerable impact. Surgical-site infection was by far the most common postoperative complication after SR.

The main limitation of this review is the difficulty in comparing a modern technique with a historical technique owing to a lack of comparative evidence and standardization of methods, inequality of reporting, and variation in follow-up. Notably, the population characteristics in terms of age, sex, and indication for surgery were similar in the two groups.

Significant variation in duration of follow-up across studies in both literature searches limited the validity of comparison. Follow-up varied from 12 to 162 months in the SR studies, and from 12 to 74 months in the VMR studies, which may have had a significant effect on the results. Variation in methods of measuring constipation and incontinence across studies, as well as varying interpretation of these methods, made comparison of studies challenging.

This review has highlighted that the recurrence rates and safety of SR and VMR are comparable; however, a robust RCT in this field is highly advocated.

*Disclosure.* The authors declare no conflict of interest.

## Supplementary material

Supplementary material is available at *BJS Open* online.

## Supplementary Material

zraa037_Supplementary_Data
