# Supplementary material for: Suture rectopexy versus ventral mesh rectopexy for complete full-thickness rectal prolapse and intussusception: systematic review and meta-analysis
Source: BJS Open. 2021 Jan 9;5(1):zraa037. doi: 10.1093/bjsopen/zraa037 (PMC7893464; doi:10.1093/bjsopen/zraa037)
Supplement: zraa037_Supplementary_Data [file zraa037_supplementary_data.zip › Tables LL Edits longer captions FINAL.docx]

Table 1: Suture rectopexy study characteristics

| Study | type of study | Population age (median/mean as stated) | percentage female | Follow-up method | Follow up in months (median unless otherwise stated) | Number of patients | MINORS score | Cochrane Collaboration Tool |
| --- | --- | --- | --- | --- | --- | --- | --- | --- |
| Benoist (2001)^15^ | retrospective | mean age 76.2 | 100 | clinical examination | 24.0 (mean) | 16 | 15/24 | - |
| Blatchford (1989)^16^ | retrospective | Average age 61 | 88.4 | office visits/telephone interviews | 28.0 | 42 | 11/16 | - |
| Briel (1997)^17^ | retrospective | Median age 71 | 87.5 | hospital records and prospective telephone interview | 67.0 | 24 | 10/16 | - |
| Bruch (1999)^18^ | prospective | mean age 62 | 94.4 | clinic appointment, continence score, anorectal manometry | 30.0 (mean) | 32 | 12/16 | - |
| Chaudhry (2010)^19^ | prospective | mean age 43.5 | 72.2 | non-specific | 12.0 | 36 | 11/16 | - |
| De Oliviera (2010)^20^ | retrospective | median age 82 | 87.5 | examination/patient complaint | 28.5 (mean) | 16 | 16/24 | - |
| Foppa (2014)^21^ | prospective | median age 62 | 97.2 | telephone interview and office appointment | 162.0 | 172 | 13/16 | - |
| Gleditsch (2018)^22^ | retrospective | median age 72 | 82.8 | interview, endoscopy and examination | 84.0 | 49 | 15/24 | - |
| Heah (2000)^23^ | retrospective | average age 72 | 88 | outpatient appointment or telephone review | 26.0 | 25 | 11/16 | - |
| Hidaka (2019)^24^ | RCT | median age 48.5 | 90 | clinical examination | 72.0 | 30 | - | 3 Unclear  4 Low Risk |
| Kellokumpu (2000)^25^   \|  \| \| --- \| | prospective | median age 57 | 91.2 | appointment at hospital and endoscopy | 24.0 | 16 | 14/24 | - |
| Kessler (1999)^26^ | retrospective | median age 51.5 | 84.4 | telephone interview | 33.0 | 28 | 9/16 | - |
| Khanna (1996)^27^ | prospective | N/A | N/A | N/A | 64.8 | 65 | 10/16 | - |
| Liyanage (2009)^28^ | prospective | median age 37 | 29.6 | outpatient appointment or telephone/postal review | 56.0 | 70 | 12/16 | - |
| Luglio (2016)^29^ | RCT | median age 68 | 100 | questionnaire, endoscopy and defecography | 12.0 | 11 | - | 5 Unclear  2 Low Risk |
| McKee (1992)^30^   \|  \| \| --- \| | RCT | mean age 70 | 50 | examination | 20.0 | 8 | - | 5 Unclear  2 Low Risk |
| Novell (1994)^31^ | RCT | median age 76 | 98.4 | outpatient appointment or telephone/postal review | 50.0 | 32 | - | 3 Unclear  3 Low Risk  1 High Risk |
| Raftopoulos (2005)^32^ | retrospective | median age 53 | 70.9 | patient data | 43.0 | 163 | 16/24 | - |
| Sahoo (2014)^33^   \|  \| \| --- \| | retrospective | mean age 42.5 | N/A | hospital records | 12.0 | 32 | 15/24 | - |
| Senapati (2013)^34^ | RCT | mean age 58 | 84.2 | clinic appointment and questionnaire | 36.0 | 35 | - | 4 Unclear  3 Low Risk |
| Wilson (2011)^35^ | prospective | median age 72 | 98.6 | telephone interview | 48.0 | 59 | 9/16 | - |
| Yasukawa (2017)^36^ | case series | mean age 72.5 | 93.3 | telephone interview | 16.3 | 15 | 10/16 | - |

Table 2: Mesh rectopexy study characteristics

| Study | | Type of study | Population age (median/mean as stated) | percentage female | Follow-up method | Follow up in months (median unless otherwise stated) | Type of mesh | Number of patients with CRP | Number of patients with IS | MINORS score | Cochrane Collaboration Tool |
| --- | --- | --- | --- | --- | --- | --- | --- | --- | --- | --- | --- |
| Albayati 2017^37^ | retrospective | | median 57 | 100.0 | questionnaire and telephone call | 22.0 | Biological | 9 | 42 | 8/16 | - |
| Benoist 2001^15^ | retrospective | | mean age 76.2 | 100.0 | clinical examination | 24.0 (mean) | N/A | 14 | - | 15/24 | - |
| Bjerke 2014^38^ | N/A | | median age 83 | 100.0 | N/A | 1.5 | Synthetic | 40 | - | 7/16 | - |
| Boons 2010^39^ | prospective | | median age 72 | 92.3 | clinic appointment and telephone call | 19.0 | Synthetic | 65 | - | 11/16 | - |
| Brunner 2018^40^ | prospective | | mean age 64.7 | 94.0 | clinical examination and questionnaire | 29.0 | Biological | 13 | - | 11/16 | - |
| Byrne 2008^41^ | prospective | | mean age 56.2 | N/A | telephone interview and contacted GP | 60.0 | Synthetic | 126 | - | 10/16 | - |
| Chandra 2016^42^ | prospective | | median age 50 | 60.0 | examination and long-term telephone consultation | 22.0 | Synthetic | 15 | - | 10/16 | - |
| Collinson 2010^43^   \|  \| \| --- \| | prospective | | median age 58 | 92.0 | outpatient clinic | 12.0 | synthetic | - | 75 | 11/16 | - |
| Consten 2015^44^ | retrospective | | mean age 55.8 | 94.6 | outpatient clinic | 40.0 | Synthetic | 242 | - | 11/16 | - |
| D'Hoore 2006^45^ | prospective | | median female age 50, median male age 32 | 91.7 | N/A | N/A | Synthetic | 109 | - | 9/16 | - |
| Emile 2017^46^ | RCT | | mean age 39.7 | 62.0 | consultation and examination | 18.0 (mean) | Synthetic | 25 | - | - | 3 Unclear  4 Low Risk |
| Faucheron 2012^47^ | prospective | | mean age 58 | 90.3 | examination | 74.0 | Synthetic | 175 | - | 12/16 | - |
| Franceschilli 2015^48^   \|  \| \| --- \| | prospective | | mean age 63 | 100.0 | outpatient clinic | 20.0 | biological | - | 98 | 13/16 | - |
| Gleditsch 2018^22^ | retrospective | | median age 72 | 82.8 | interview, endoscopy and examination | 29.0 | Biological or synthetic | 22 | - | 16/24 | - |
| Gosselink 2015^49^ | prospective | | median age 63 for CRP, median age 59 for intussusception | 92.7 | questionnaire and outpatient clinic | 12.0 | Synthetic | 41 | 50 | 10/16 | - |
| Hidaka 2019^24^ | RCT | | 56.5 median age | 91.2 | clinical examination | 72.0 | N/A | 34 | - | - | 3 Unclear  4 Low Risk |
| Hiltunen 1991^50^ | prospective | | mean age 53 | 81.5 | outpatient clinic | 36.0 | Synthetic | 54 | - | 12/16 | - |
| Lechaux 2005^51^ | retrospective | | median age 53 | 91.7 | clinical review and postal questionnaire | 36.0 | Synthetic | 35 | - | 9/16 | - |
| Luglio 2017^29^ | RCT | | median age 68 | 100.0 | questionnaire, endoscopy and defecography | 12.0 | N/A | 20 | - | - | 5 Unclear  2 Low Risk |
| Madbouly 2017^52^ | retrospective | | mean age 55 | 81.1 | clinical review and postal questionnaire | 46.0 (mean) | N/A | 41 | - | 18/24 | - |
| Maggiori 2013^53^ | prospective | | mean age 64 | 87.9 | examination or telephone consultation | 42.0 | Synthetic | 20 | - | 10/16 | - |
| Mantoo 2013^54^ | prospective | | mean age 62 | N/A | outpatient clinic | 16.0 | Synthetic | 23 | - | 19/24 | - |
| Mehmood 2014^55^ | prospective | | median age 59 | 94.1 | questionnaire | 12.0 | Biological | 34 | - | 17/24 | - |
| Ogilvie 2014^56^ | prospective | | mean age 72.3 | 100.0 | clinic/examination | 16.0 | Synthetic | 33 | - | 16/24 | - |
| Owais 2014^57^ | prospective | | median age 34.5 | 0.0 | questionnaire | 42.0 | Mostly synthetic | 18 | 50 | 9/16 | - |
| Portier 2011^58^ | prospective | | mean age 60.6 | 100.0 | outpatient clinic, examination and questionnaire | 22.0 (mean) | Synthetic | - | 40 | 9/16 | - |
| Raftopoulos 2005^32^   \|  \| \| --- \| | retrospective | | median age 53 | 70.9 | patient data | 43.0 | Synthetic | 125 | - | 16/24 | - |
| Randall 2014^59^ | prospective | | median age 69 | 87.4 | appointment | 29.0 | Synthetic | 190 | - | 11/16 | - |
| Tsunoda 2016^60^ | prospective | | median age 76 | 100.0 | questionnaires and proctography | 26.0 | Synthetic | - | 44 | 9/16 | - |
| Tsunoda 2019^61^ | retrospective | | median age 80 | 89.6 | Outpatient clinic/phone interview/mail questionnaire | 49.0 | Synthetic | 58 | - | 10/16 | - |
| Wahed 2012^62^ | prospective | | median age 62 | 95.4 | examination and proctogram | 12.0 | Biological | 27 | - | 11/16 | - |

Table 3: Recurrences according to surgical approach

| Author (Year) | Number of patients | Number of recurrences (%) |
| --- | --- | --- |
| Suture Rectopexy |  |  |
| Benoist (2001) | 16 | 0 (0) |
| Blatchford (1989) | 42 | 1 (2.4) |
| Briel (1997) | 24 | 0 (0) |
| Bruch (1999) | 32 | 0 (0) |
| Chaudhry (2010) | 36 | 1 (2.8) |
| De Oliviera (2010) | 16 | 2 (12.5) |
| Foppa (2014) | 172 | 30 (17.4) |
| Gleditsch (2018) | 49 | 15 (30.6) |
| Heah (2000) | 25 | 0 (0) |
| Hidaka (2019) | 30 | 7 (23.3) |
| 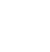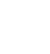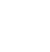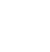Kellokumpu (2000) | 16 | 2 (12.5) |
| Kessler (1999) | 28 | 2 (7.1) |
| Khanna (1996) | 65 | 0 (0) |
| Liyanage (2009) | 70 | 5 (7.1) |
| Luglio (2016) | 11 | 3 (27.3) |
| 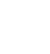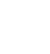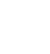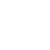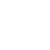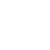Mckee (1992) | 8 | 0 (0) |
| Novell (1994) | 32 | 1 (3.1) |
| Raftopoulos (2005) | 163 | 1 (0.1) |
| Sahoo (2014) | 32 | 0 (0) |
| Senapati (2013) | 35 | 9 (25.7) |
| Wilson (2011) | 59 | 6 (10.2) |
| Yasukawa (2017) | 15 | 1 (6.7) |
| Total | 976 | 84 (8.6) |
| Ventral Mesh Rectopexy |  |  |
| Recurrence of Complete Rectal Prolapse |  |  |
| Albayati (2017) | 9 | 1 (11.1) |
| Benoist (2001) | 14 | 0 (0) |
| Bjerke (2014) | 40 | 2 (5.0) |
| Boons (2010) | 65 | 1 (1.5) |
| Brunner (2018) | 13 | 1 (7.7) |
| Byrne (2008) | 126 | 5 (4.0) |
| Chandra (2016) | 15 | 0 (0) |
| Consten (2015) | 242 | 13 (5.4) |
| D’Hoore (2006) | 109 | 4 (3.7) |
| Emile (2017) | 25 | 2 (8.0) |
| Faucheron (2012) | 175 | 2 (1.1) |
| Gleditsch (2018) | 22 | 3 (13.6) |
| Gosselink (2015) | 41 | 1 (2.4) |
| Hidaka (2019) | 34 | 3 (8.8) |
| Hiltunen (1991) | 54 | 1 (1.9) |
| Lechaux (2005) | 35 | 1 (2.9) |
| Luglio (2017) | 20 | 1 (5.0) |
| Madbouly (2017) | 41 | 1 (2.4) |
| Maggiori (2013) | 20 | 0 (0) |
| Mantoo (2013) | 23 | 2 (8.7) |
| Mehmood (2014) | 34 | 0 (0) |
| Ogilvie (2014) | 33 | 5 (15.2) |
| Owais (2014) | 18 | 0 (0) |
| Raftopoulos (2005) | 125 | 9 (7.2) |
| Randall (2014) | 190 | 1 (0.5) |
| 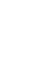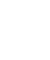Tsunoda (2019) | 58 | 1 (1.7) |
| Wahed (2012) | 27 | 1 (3.7) |
| Total | 1605 | 60 (3.7) |
| Recurrence of Intussusception |  |  |
| Albayati (2017) | 42 | 2 (4.8) |
| Collinson (2010) | 75 | 4 (5.3) |
| Franceschilli (2015) | 98 | 14 (14.3) |
| Gosselink (2015) | 50 | 3 (6.0) |
| Owais (2014) | 50 | 0 (0) |
| Portier (2011) | 40 | 1 (2.5) |
| Tsunoda (2016) | 44 | 15 (34.1) |
| Total | 402 | 39 (9.7) |

Table 4: Recurrence rates following suture and mesh rectopexy showing a significant increase in recurrence of CRP following SR compared to VMR

|  | Suture Rectopexy | | Ventral mesh rectopexy | |
| --- | --- | --- | --- | --- |
| Indication | CRP (n=976) | IS (n=0) | CRP (n=1605) | IS (n=402) |
| Recurrence | 84 (8.6%) | - | 60 (3.7%) | 39 (9.7) |

*CRP = complete rectal prolapse, IS = intussusception*

*p = 0.000000176 for SR versus VMR for CRP*

*Table 5: Comparison between biological and synthetic mesh for mesh rectopexy showing no significant difference between biological and synthetic mesh for CRP or IS*

| Type of mesh | Number of studies | | Number of patients | | Number of recurrences (%) | | |
| --- | --- | --- | --- | --- | --- | --- | --- |
| Indication | **CRP** | **IS** | **CRP** | **IS** | **CRP** | **IS** | **CRP and IS** |
| Biological | 5 | 2 | 97 | 140 | 4 (4.1) | 16 (11.4) | 20/237 (8.4) |
| Synthetic | 17 | 4 | 1362 | 209 | 49 (3.6) | 23 (11.0) | 72/1571 (4.6) |
|  |  |  |  |  | *p = 0.789* | *p = 0.902* |  |

*CRP = complete rectal prolapse, IS = intussusception*

Table 6: Constipation and incontinence in different studies showing overall improvements of incontinence in both groups whilst improvement of constipation was seen more in the VMR group.

|  | Method of measuring incontinence | Statistically significant improvement | Method of measuring constipation | Statistically significant improvement |
| --- | --- | --- | --- | --- |
| Suture Rectopexy (CRP) |  |  |  |  |
| Benoist (2001) | Raw figures | Not stated | Raw figures | Not stated |
| Blatchford (1989) | Graded 0-4 and raw figures | Yes | Raw figures | No, significantly worse constipation |
| Briel (1997) | Browning and Parks | Unclear | N/A | N/A |
| Bruch (1999) | Luebeck continence score | Yes | Raw figures | Yes, but includes some patients who had resection rectopexy |
| Chaudhry (2010) | Browning and Parks | Yes | Raw figures | Not stated but 9/15 patients improved |
| De Oliviera (2010) | Wexner Score | Not stated but 9/11 patients improved | N/A | N/A |
| Foppa (2014) | Wexner Score | Yes | Wexner Score | No |
| Gleditsch (2018) | N/A | N/A | N/A | N/A |
| Heah (2000) | Browning and Parks | Yes | Raw figures | No |
| Hidaka (2019) | Cleveland Clinic Incontinence Score | Not stated | Cleveland Clinic Constipation Score, Patient Assessment of Constipation Quality of Life questionnaire (PAC-QOL) and The Patient Assessment of Constipation symptom score (PAC-SYM) | Not stated |
| Kellokumpu (2000)   \|  \| \| --- \| | Browning and Parks | Yes | Numerical symptom score | Yes |
| Kessler (1999) | N/A | N/A | N/A | N/A |
| Khanna (1996) | Raw figures | Not stated but 12/16 patients improved | Raw figures | Not stated but 5/6 patients improved |
| Liyanage (2009) | Wexner Score and Browning and Parks | Yes, but includes some patients who had resection rectopexy | Rome II Criteria | Not stated |
| Luglio (2016) | Wexner Score | Not stated | Wexner Score | Not stated |
| McKee (1992)   \|  \| \| --- \| | Saline solution infusion test (Raw figures) | Not stated but only 1/5 patients had post-operative incontinence | Raw figures | No |
| Novell (1994) | Browning and Parks/Raw figures | Not stated but 7/10 regained continence to solid and liquid | N/A | N/A |
| Raftopoulos (2005) | N/A | N/A | N/A | N/A |
| Sahoo (2014)   \|  \| \| --- \| | Wexner Score | Not stated but 19/21 patients improved | Wexner Score | Not stated but 11/18 patients improved |
| Senapati (2013) | Vaizey Score | Yes | N/A | N/A |
| Wilson (2011) | N/A | N/A | N/A | N/A |
| Yasukawa (2017) | N/A | N/A | Raw figures | Not stated, 4/10 patients improved |
| Ventral Mesh Rectopexy (CRP and IS) |  |  |  |  |
| Albayati (2017) (CRP) | Raw figures | No | Raw figures | No |
| Albayati (2017) (IS) | Raw figures | Yes | Raw figures | Yes |
| Benoist (2001) | Raw figures | Not stated | Raw figures | Not stated |
| Bjerke (2014) (CRP) | Wexner Score | Yes | Laxatives use (Raw figures) | No |
| Boons (2010) (CRP) | Faecal Incontinence Severity Index (FISI) | Yes | Wexner Score | Yes |
| Brunner (2018) (CRP) | Cleveland Clinic Incontinence Score (CCIS) | Yes | CCIS | Yes |
| Byrne (2008) (CRP) | St Mark’s Incontinence Score | Yes | Visual analog constipation score and perceived change (raw figures) | No |
| Chandra (2016) (CRP) | FISI | Yes | Wexner Score | Yes |
| Collinson (2010) (IS) | FISI | Yes | Wexner Score | Yes |
| Consten (2015) (CRP) | Browning and Parks | Yes, but includes patients with IS/symptomatic rectocele not included in recurrence data | Rome II Criteria | Not stated but 50/82 improved |
| D’Hoore (2006) (CRP) | N/A | N/A | N/A | N/A |
| Emile (2017) (CRP) | Wexner Score | Yes | Wexner Score | Not stated but large improvement in Wexner score |
| Faucheron (2012) (CRP) | N/A | N/A | N/A | N/A |
| Franceschilli (2015) (IS) | FISI | Yes | Wexner Score | Yes |
| Gleditsch (2018) (CRP) | N/A | N/A | N/A | N/A |
| Gosselink (2015) (CRP) | FISI | Yes | Wexner Score | Yes |
| Gosselink (2015) (IS) | FISI | Yes | Wexner Score | Yes |
| Hidaka (2019) | CCIS | Not stated | Cleveland Clinic Constipation Score, Patient Assessment of Constipation Quality of Life questionnaire (PAC-QOL) and The Patient Assessment of Constipation symptom score (PAC-SYM) | Not stated |
| Hiltunen (1991) (CRP) | Raw figures | Yes | N/A | N/A |
| Lechaux (2005) (CRP) | Wexner Score | No | Wexner Score | Not stated |
| Luglio (2017) | Wexner Score | Not stated | Wexner Score | Not stated |
| Madbouly (2017) (CRP) | Wexner Score | Yes | Wexner Score | Yes |
| Maggiori (2013) (CRP) | Wexner Score | Yes | Rome II Criteria | Not stated by 13/18 patients had improvement |
| Mantoo (2013) (CRP) | Wexner Score | Unclear | Obstructive Daefacation Syndrome (ODS) Score | Not stated by improvement in mean score |
| Mehmood (2014) (CRP) | FISI | Yes | Wexner Score | Yes |
| Ogilvie (2014) (CRP) | CCIS | Not stated but large improvement in mean CCIS scores | N/A | N/A |
| Owais (2014) (IS and CRP) | CCIS | Yes | ODS Score | Yes |
| Portier (2011) (IS) | CCIS | Yes | Raw figures | Not stated but 13/20 improved |
| Raftopoulos (2005) | N/A | N/A | N/A | N/A |
| Randall (2014) (CRP) | CCIS | Yes | N/A | N/A |
| Tsunoda (2016) (IS) | FISI | Yes | Constipation Scoring System (CSS) | Yes |
| Tsunoda (2019) (CRP) | FISI | Yes | CSS | Yes |
| Wahed (2012) (CRP) | Wexner Score | Yes | Wexner Score | Yes |

*CRP = complete rectal prolapse, IS = intussusception*

Table 7: Constipation and incontinence in comparative studies

| Study | Method of measuring incontinence | Incontinence results | Method of measuring constipation | Constipation results |
| --- | --- | --- | --- | --- |
| Benoist (2001) | Raw figures | No significant difference | Raw figures | No stated however a similar worsening in constipation following VMR and SR was reported |
| Hidaka (2019) | Cleveland Clinic Incontinence Score | No significant difference | Cleveland Clinic Constipation Score, Patient Assessment of Constipation Quality of Life questionnaire (PAC-QOL) and The Patient Assessment of Constipation symptom score (PAC-SYM) | VMR statistically better than SR in all parameters |
| Luglio (2016) | Wexner Score | VMR statistically better than SR | Wexner Score | VMR statistically better than SR however some resection rectopexy patients included in SR arm |

Table 8: Summary of complications by procedure showing no significant difference in complication rates between procedures

| Complication | Suture rectopexy (n = 616) (%) | Mesh rectopexy (n = 1232) (%) |
| --- | --- | --- |
| Atelectasis | 0 | 1 (0.1) |
| Atrial fibrillation | 1 (0.2) | 0 |
| Bladder injury | 0 | 1 (0.1) |
| Bleeding from port-site | 1 (0.2) | 0 |
| Deep vein thrombosis | 4 (0.6) | 0 |
| Enterocutaneous fistula | 0 | 0 |
| Faecal impaction | 0 | 1 (0.1) |
| Fluid overload | 0 | 1 (0.1) |
| Haematoma | 1 (0.2) | 10 (0.8) |
| Hypertension | 1 (0.2) | 0 |
| Incisional/port site hernia | 3 (0.5) | 7 (0.6) |
| Infective diarrhoea | 2 (0.3) | 0 |
| Intestinal obstruction | 4 (0.6) | 2 (0.2) |
| Lumbar discitis | 0 | 1 (0.1) |
| Myocardial infarction | 0 | 1 (0.1) |
| Non-specific bleeding | 1 (0.2) | 1 (0.1) |
| Non-specific infection | 0 | 2 (0.2) |
| Pain | 0 | 6 (0.5) |
| Pelvic abscess | 2 (0.3) | 0 |
| Pelvic collection | 1 (0.2) | 0 |
| Perforated bowel | 2 (0.3) | 3 (0.2) |
| Peritonitis | 1 (0.2) | 0 |
| Pneumonia | 3 (0.5) | 3 (0.2) |
| Presacral vein injury | 2 (0.3) | 0 |
| Prolonged ileus | 1 (0.2) | 12 (1.0) |
| Pulmonary oedema | 0 | 0 |
| Respiratory failure | 0 | 0 |
| Retrograde ejaculation | 0 | 0 |
| Sphincterismus | 0 | 0 |
| Subcutaneous emphysema | 1 (0.2) | 3 (0.2) |
| Surgical site infection | 12 (1.8) | 5 (0.4) |
| Upper GI bleed | 0 | 0 |
| Ureteric injury | 2 (0.3) | 1 (0.1) |
| Urinary incontinence | 0 | 2 (0.2) |
| Urinary retention | 6 (0.9) | 4 (0.3) |
| Urinary tract infection | 3 (0.5) | 29 (2.4) |
| Wound abscess | 0 | 1 (0.1) |
| Total | **54 (8.8%)** | **97 (7.9%)** (p = 0.509) |

*Table 9. Characteristics of studies included for meta-analysis*

| Author, year | Study design | Study period | Journal | Number of patients with suture rectopexy without resection | Number of patients with ventral mesh rectopexy | Comparators | Inclusion | Exclusion | Method of measuring recurrence | Outcome measures | Length of follow-up period in months (median unless stated otherwise) |
| --- | --- | --- | --- | --- | --- | --- | --- | --- | --- | --- | --- |
| Benoist et al., 2001 | Retrospective, observational | 1993-1995 | The American Journal of Surgery | 16 | 14 | Mesh rectopexy versus suture rectopexy with and without sigmoid resection | Patients who had surgery for full-thickness rectal prolapse | Patients who had a hand assisted procedure | Clinical examination or long-term telephone interview | Complcations, constipation, incontinence, recurrence | 24 (mean) |
| Gleditsch et al., 2018 | Retrospective, observational | 1998-2017 | Langenbeck’s Archives of Surgery | 49 | 22 | Laparoscopic posterior sutured rectopexy versus ventral mesh rectopexy | Patients who had surgery for external rectal prolapse | Patients with internal rectal prolapse | Clinical examination and endoscopy | Complications, recurrence | 84 for suture rectopexy, 29 for ventral mesh rectopexy |
| Hidaka et al., 2019 | Randomised control trial | 2006-2014 | EClinicalMedicine | 34 | 38 | Laparoscopic posterior sutured rectopexy versus ventral mesh rectopexy | Patients with rectal prolapse | N/A | Clinical examination and questionnaires | constipation symptom score (PAC-SYM), quality of life score (PAC-QoL), obstructed defecation score (ODS), Cleveland clinic constipation and incontinence scores (CCCS, CCIS), prolapse recurrence, mesh complications | 6.1 years (73.2 months) |
| Luglio et al., 2016 | Randomised control trial | 2013-2015 | Aging Clinical and Experimental Research | 11 | 20 | Ventral mesh rectopexy versus suture rectopexy | obstructed defecation syndrome, persistent bleeding, full-thickness rectal prolapse, squeeze pressure >60 mmHg | N/A | questionnaire, endoscopy and defecography | Rome III criteria, Wexner Incontinence score and Wexner constipation score  endoscopy and defecography | 12 |
| Raftopoulos et al., 2005 | Retrospective, observational | 1979-2001 | Diseases of the Colon and Rectum | 122 | 117 | Mobilization-only, mobilization-resection-pexy, or mobilization-pexy. Means of access: open or laparoscopic. Rectopexy method: suture or mesh. | Patients who had abdominal surgery for full thickness rectal prolapse | Patients were excluded if follow up was not achieved | physical examination in the outpatient clinic or by telephone interview | Recurrence | 43 |
