# Supplementary material for: Suture rectopexy versus ventral mesh rectopexy for complete full-thickness rectal prolapse and intussusception: systematic review and meta-analysis
Source: BJS Open. 2021 Jan 9;5(1):zraa037. doi: 10.1093/bjsopen/zraa037 (PMC7893464; doi:10.1093/bjsopen/zraa037)
Supplement: zraa037_Supplementary_Data [file zraa037_supplementary_data.zip › Supplement Tables.docx]

**Table S1 Newcastle–Ottawa Scale assessing the quality of non-randomized studies**

| Study | Selection | | | Comparability | Outcome | | Total |
| --- | --- | --- | --- | --- | --- | --- | --- |
|  | **Representativeness of exposed cohort (*)** | **Selection of non-exposed cohort (*)** | **Ascertainment of exposure (*)** | **(**)** | **Assessment of outcome (*)** | **Adequacy of follow-up (*)** | **(7*)** |
| Benoist et al., 2001 | - | * | * | * | * | - | 4/7 |
| Gleditsch et al., 2018 | * | * | * | * | * | * | 7/7 |
| Raftopoulos et al., 2005 | * | * | * | ** | - | * | 6/7 |

**Table S2 Cochrane Collaboration tool assessing the quality of randomized studies**

| Study | Selection Bias | | Performance Bias | Detection bias | Attrition bias | Reporting bias | Other |
| --- | --- | --- | --- | --- | --- | --- | --- |
|  | **Random sequence generation** | **Allocation concealment** | **Blinding of participants and personnel** | **Blinding of outcome assessment** | **Incomplete outcome data** | **Selective reporting** |  |
| Emile et al., 2017 | Low risk | Low risk | Unclear | Unclear | Low risk | Low risk | Unclear |
| Hidaka et al., 2019 | Unclear | Unclear | Low risk | Low risk | Low risk | Low risk | Unclear |
| Luglio et al., 2016 | Unclear | Unclear | Unclear | Unclear | Low risk | Low risk | Unclear |
| McKee et al., 1992 | Unclear | Unclear | Unclear | Unclear | Low risk | Low risk | Unclear |
| Novell et al., 1994 | Low risk | Low risk | Unclear | Unclear | High risk* | Low risk | Unclear |

**12/63 patients died during follow-up*
